# Commentary: The effectiveness of repetitive transcranial magnetic stimulation for post-stroke dysphagia: A systematic review and meta-analysis

**DOI:** 10.3389/fnhum.2022.1018885

**Published:** 2022-12-01

**Authors:** Ting Li, Xiao-Yan Duan, Xiu-Mei Zou, Xi Huang, Yu-Juan Kang, Ming-Zhu Sun

**Affiliations:** ^1^School of Nursing, Shaanxi University of Chinese Medicine, Xianyang, China; ^2^Outpatient Department, Affiliated Hospital of Shaanxi University of Chinese Medicine, Xianyang, China

**Keywords:** dysphagia, transcranial magnetic stimulation, post-stroke, meta-analysis, commentary

## Introduction

We read the study of Wen et al. (2022), with great interest and appreciate the authors' work in this field, whose findings suggest that high frequency repetitive transcranial magnetic stimulation (rTMS) has a more significant effect than that of low-frequency rTMS on post-stroke dysphagia. However, we believe the authors may have overestimated their findings, and some issues exist that may question the effectiveness of the meta-analysis, which is worthy of discussion. Below we expand on three areas in which the efficacy of the meta-analysis may have been compromised.

## Insufficient inclusion of studies

Despite searching relevant databases, Wen et al. missed several randomized controlled trials that met the inclusion criteria and were included in relevant databases such as PubMed and Web of Science. For instance, Park et al. (2017) investigated the effects of bilateral 10 Hz rTMS on patients with post-stroke dysphasia using the Clinical Dysphagia Scale (CDS), Dysphagia Outcome and Severity Scale (DOSS), Videofluoroscopic Dysphagia Scale (VDS) and Penetration Aspiration Scale (PAS) as outcome measures. Additionally, Ünlüer et al. randomized 28 stroke patients into an rTMS group and a control group and evaluated the PAS. Both groups received conventional swallowing rehabilitation therapy for 4 weeks, and the rTMS group additionally had low frequency rTMS of 1 Hz applied to the unaffected hemisphere during the last week (Ünlüer et al., 2019). Finally, Tarameshlu et al. (2019) randomly divided 18 patients into three groups: traditional swallowing rehabilitation therapy, 1 Hz rTMS therapy, and combination therapy, and used the Mann Assessment of Swallowing Ability (MASA) and the Functional Oral Intake Scale (FOIS) as outcome measures. Potentially, these three randomized clinical trials (RCTs) were not adopted in the meta-analysis may be due to the way in which the data is presented within these studies. In the study by Park et al., the data was presented in the form of chart. In the study by Taramechlu et al., the data is presented in the form of an interquartile range, while Wen et al. choose the data presented in the text to be presented in the form of Mean ± SD ($\bar{x}\pm s$). However, the study of Ünlüer et al., fully conforms to Wen's nanofiltration standard, but was left out. Therefore, it is suggested that Wen et al. should more clearly refine the criteria for inclusion and exclusion [for example, exclude other types of data, and only limit the type of data included in the study to be explicit ($\bar{x}\pm s$)]. In addition, in order to avoid missing matching articles, the screening process should be more carefully and strictly implemented.

## Inappropriate data extraction and analysis

The results of the meta-analysis by Wen et al. showed high heterogeneity (Chi^2^ = 77.28, I^2^ = 78%). After the authors performed a random effects model, the heterogeneity within the results of the meta-analysis was still high. According to the methodology of the meta-analysis, the change values before and after intervention were re-extracted one by one. In the study of Cai et al. (2019), the authors observed the effect of high-frequency repetitive transcranial magnetic stimulation (rTMS) on dysphagia after stroke in the representative area of the mylohyoid muscle cortex of bilateral cerebral hemispheres. SSA, DOSS and PAS were used as outcome measures. The SSA score ranges from 18 to 46, and the higher the score, the more severe the dysphagia. We extracted SSA change values of 9.67 ± 0.88 for the bilateral stimulation group, 6.03 ± 1.09 for the unilateral group, and 2.93 ± 1.34 for the control group, while Wen et al. extracted SSA change values of 2.65 ± 0.27 for the bilateral group, 1.6 ± 0.24 for the unilateral group, and 0.65 ± 0.32 for the control group. We speculate that Wen et al. may have extracted incorrect data. In the Cochrane Handbook (Higgins et al., 2019), it is explained that the comparison of the measured values after intervention and the difference between pre-and post-intervention have the same analytical value in theory. Therefore, post-intervention measurements were chosen to be re-analyzed.

## Unclear criteria of outcome indicators

In the meta-analysis by Wen et al., the category of PAS values that were measured were not specified, which could increase the measurement uncertainty and the error of the data. Therefore, when re-extracting the data of PAS, we selected liquid measurement if both liquid and semi-solid values were included in the study (Lim et al., 2014; Ünlüer et al., 2019). Thin liquid values were selected if it was thin liquid, thick liquid and semisolid was used. This was done because, when swallowing, the thin liquid is more likely to flow too quickly into the airway below the vocal cords, leading to aspiration and increasing the risk of aspiration pneumonia (Winstein et al., 2016; McCurtin et al., 2020).

Based on the above problems, we added three randomized controlled trials (Park et al., 2017; Tarameshlu et al., 2019; Ünlüer et al., 2019) to the meta-analysis that met the inclusion and exclusion criteria. The study of Park et al. provided the raw data in graphs, from which we extracted the PAS values using Origin software. Using the method of Luo et al. (2017) the interquartile interval data in the article by Taramechlu et al. are converted into mean ± standard deviation. Wen's inclusion criteria indicated that RCT with two or more interventions should be included regardless of the sample size. Therefore, we included the study of Ünlüer et al. Data extraction and analysis were performed per the criteria in the meta-analysis by Wen et al.: (1) Re-extract representative end values such as PAS, SSA, DD and FOIS (all expressed as Mean ± SD). For the FOIS scale, better feeding function is indicated by higher scores. However, this is opposite from the DD, PAS and SSA scales. Therefore, according to the Cochrane Handbook (Higgins et al., 2019), the FOIS scores were multiplied by −1 to ensure that all units of measurement pointed in the same direction. (2) Finally, the liquid value was used for the PAS scores.

**Figure 1:**
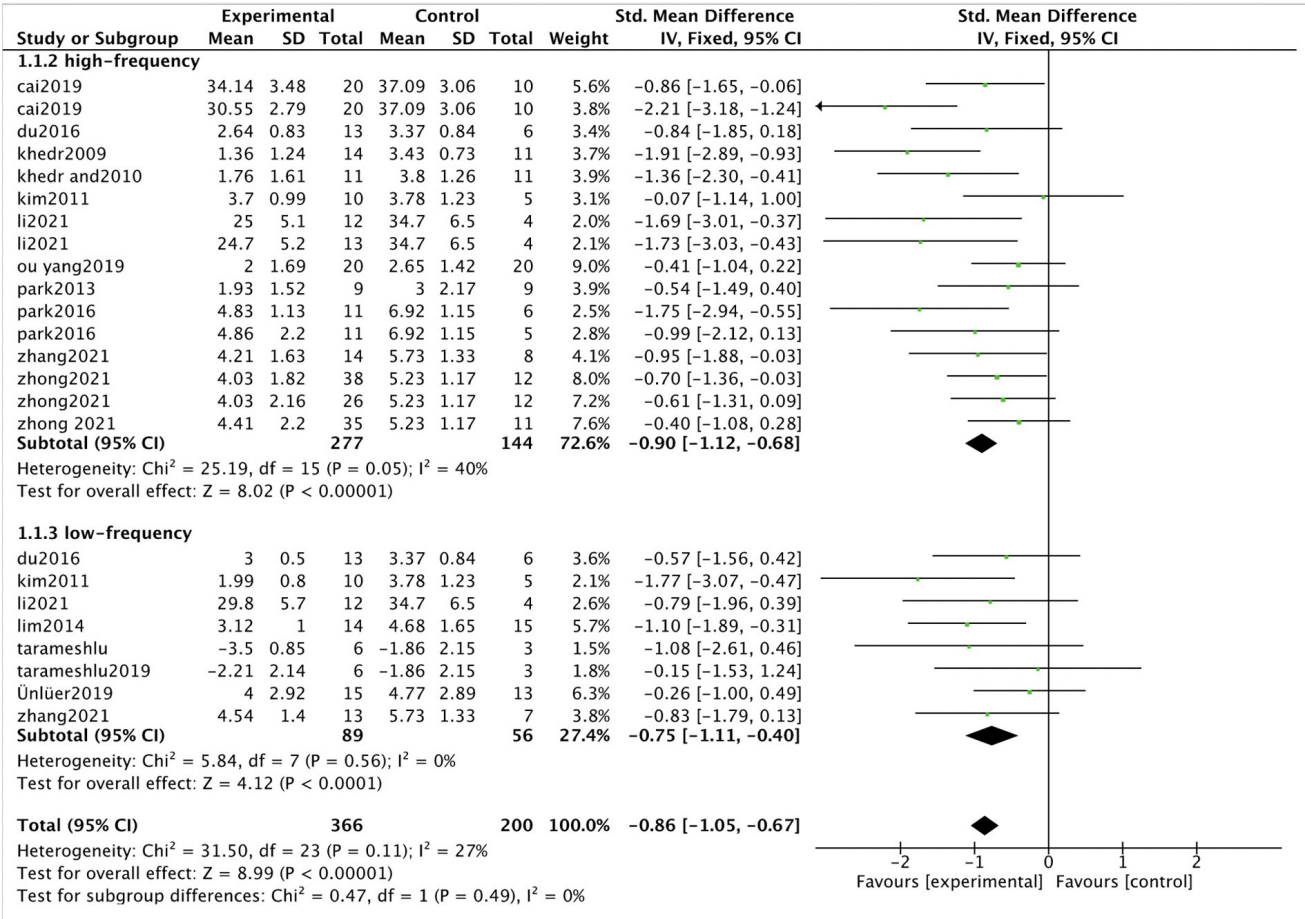
Forest plot of value post-intervention to the checkpoint for swallowing function. SMD, Standardized mean difference; CI, confidence interval; N, number of participants.

We used 5.4 to analyze and redraw all the studies data. The heterogeneity was as follows: Chi^2^ = 31.50, I^2^ = 27%, and fixed effects models were applied with effect size of [SMD = −0.86 (−1.05, −0.67), *p* < 0.001] (Figure 1), compared with high heterogeneity of I^2^ = 78% and effect size of [SMD = 2.15 (1.61, 2.70), (*p* < 0.001)] in the study of Wen et al. The heterogeneity of this study was significantly lower than that of Wen et al., and the effect size was smaller. Further evidence from various studies suggests that, although rTMS does have some efficacy in the treatment of dysphagia, the effect size may not be as high as reported by Wen et al. For example, a similar meta-analysis by Liao et al. (2017) showed that rTMS significantly improved swallowing function after stroke [SMD = 1.24 (0.67, 1.81), *p* < 0.01]. Additionally, Cheng et al. (2021), evaluated the efficacy of nerve stimulation for dysphagia after stroke, including rTMS, transcranial direct current stimulation (tDCS), and pharyngeal electrical stimulation (PES). The effect of rTMS was most significant [SMD = 0.73(0.49, 0.98), *p* < 0.001)]. Twelve randomized controlled studies were included in Qiao's meta-analysis (Qiao et al., 2022), and the total effect value showed that rTMS had a positive effect on swallowing in patients [SMD = −0.67 (−0.88, −0.46), *p* < 0.001]. The study showed (Li et al., 2022) that swallowing after stroke was significantly improved after immediate intervention with rTMS [SMD = 0.85 (0.45, 1.24), *p* < 0.001]. The study by Zhu and Gu (2022) showed that rTMS increased swallowing function compared with the sham stimulation group [SMD = 1.08 (0.37, 1.80), I^2^ = 81.2%, *p* < 0.001]. Xie et al. (2022a,b) reported two articles about the influence of rTMS for dysphasia after stroke, and the effect value showed [SMD = −0.76 (−1.07, −0.46), (*p* < 0.001)] and [SMD = −0.87 (−1.22, −0.52), (*p* < 0.001)], respectively.

Furthermore, we performed subgroup analysis according to transcranial magnetic frequency and found that the high frequency group effect value was [SMD = −0.90 (−1.12, −0.68), *p* < 0.001, *I*^2^ = 40%] and the low-frequency group was [SMD = −0.75 (−1.11, −0.40), *p* < 0.001, *I*^2^=0%]. Compared with the control group, both the high and low-frequency groups could significantly improve the swallowing function of patients with dysphagia. However, there was no significant difference between the low-frequency group and the high-frequency group (*p* = 0.49). The results of this study are the same as findings from similar studies (Du et al., 2016; Qiao et al., 2022; Xie et al., 2022b). In the meta-analysis by Wen et al., the effect size of the high-frequency group was [SMD = 2.50 (1.84, 3.16), *p* < 0.001, *I*^2^ = 79%] and that of the low-frequency group was [SMD = 1.26 (0.61, 1.90), *P* < 0.001, *I*^2^ = 45%]. Significant differences in subgroup analyses (high and low-frequency groups) were reported by Wen et al. (*P* = 0.008). Because Wen et al. may have extracted the data incorrectly, the conclusion may be inaccurate. In addition, the heterogeneity in subgroup analysis was high (*I*^2^ = 85.7%), and high heterogeneity may indicate incorrect data extraction and input. Wen et al. may need to check the data again to give a reasonable explanation for heterogeneity.

Overall, the meta-analysis by Wen et al. indicated a difference in the efficacy of low and high frequency rTMS in the treatment of dysphasia post-stroke. However, we have pointed out numerous considerations. First, there seems to study missing from the meta-analysis. Second, there was unclear selection of data indicators and incorrect data extraction, leading to large data heterogeneity, which is also a major limitation on the interpretation of the results. Readers should be cautious about the results of this meta-analysis. Finally, thanks again for the authors' contribution to the research, and I hope to provide some help and reference for their future work.

## Author contributions

TL, XH, and X-MZ conceived the idea and wrote the manuscript. M-ZS, Y-JK, and X-YD performed the data analysis. All authors examined the finished manuscript. All authors contributed to the article and approved the submitted version.

## Conflict of interest

The authors declare that the research was conducted in the absence of any commercial or financial relationships that could be construed as a potential conflict of interest.

## Publisher's note

All claims expressed in this article are solely those of the authors and do not necessarily represent those of their affiliated organizations, or those of the publisher, the editors and the reviewers. Any product that may be evaluated in this article, or claim that may be made by its manufacturer, is not guaranteed or endorsed by the publisher.

## References

[B1] Cai Q. Yang X. Sun W. Xu L. Liu J. Ma M. (2019). Efficacy of bilateral high-frequency repetitive transcranial magnetic stimulation in the treatment of post-stroke dysphagia. Chin. J. Phys. Med. Rehabil. 41, 932–934. 10.3760/cma.j.issn.0254.2019.12.01327593709

[B2] Cheng I. Sasegbon A. Hamdy S. (2021). Effects of neurostimulation on poststroke dysphagia: a synthesis of current evidence from randomized controlled trials. Neuromodulation 24, 1388–1401. 10.1111/ner.13327 33301231 PMC9292042

[B3] Du J. Yang F. Liu L. Hu J. Cai B. Liu W.. (2016). Repetitive transcranial magnetic stimulation for rehabilitation of poststroke dysphagia: a randomized, double-blind clinical trial. Clin Neurophysiol 127, 1907–1913. 10.1016/j.clinph.2015.11.045 26778719

[B4] Higgins J. P. Thomas J. Chandler J. Cumpston M. Li T. Page M. J.. (2019). Cochrane Handbook for Systematic Reviews of Interventions. New York, NY: John Wiley and Sons.

[B5] Li H. Li L. Zhang R. Huang X. Lin J. Liu C.. (2022). Effectiveness of repetitive transcranial magnetic stimulation on poststroke dysphagia: a meta-analysis of randomized-controlled trials. Int. J. Rehabil. Res. 45, 109–117. 10.1097/mrr.0000000000000517 35089877

[B6] Liao X. Xing G. Guo Z. Jin Y. Tang Q. He B.. (2017). Repetitive transcranial magnetic stimulation as an alternative therapy for dysphagia after stroke: a systematic review and meta-analysis. Clin. Rehabil 31, 289–298. 10.1177/0269215516644771 27113337

[B7] Lim K. B. Lee H. J. Yoo J. Kwon Y. G. (2014). Effect of low-frequency rTMS and NMES on subacute unilateral hemispheric stroke with dysphagia. Ann. Rehabil. Med. 38, 592–602. 10.5535/arm.2014.38.5.592 25379488 PMC4221387

[B8] Luo D. Wang X. Liu J. Tong T. (2017). How to estimate the sample mean and standard deviation from the sample size, median, extremes or quartiles? Chin. J. Evid. Based Med. 17, 1350–1356. 10.7507/1672-2531.201706060

[B9] McCurtin A. Boland P. Kavanagh M. Lisiecka D. Roche C. Galvin R. (2020). Do stroke clinical practice guideline recommendations for the intervention of thickened liquids for aspiration support evidence based decision making? A systematic review and narrative synthesis. J. Eval. Clin. Pract. 26, 1744–1760. 10.1111/jep.13372 32083782 PMC7687236

[B10] Park E. Kim M. S. Chang W. H. Oh S. M. Kim Y. K. Lee A.. (2017). Effects of bilateral repetitive transcranial magnetic stimulation on post-stroke dysphagia. Brain Stimul. 10, 75–82. 10.1016/j.brs.2016.08.005 27593709

[B11] Qiao J. Ye Q. P. Wu Z. M. Dai Y. Dou Z. L. (2022). The effect and optimal parameters of repetitive transcranial magnetic stimulation on poststroke dysphagia: a meta-analysis of randomized controlled trials. Front. Neurosci. 16, 845737. 10.3389/fnins.2022.845737 35573312 PMC9095943

[B12] Tarameshlu M. Ansari N. N. Ghelichi L. Jalaei S. (2019). The effect of repetitive transcranial magnetic stimulation combined with traditional dysphagia therapy on poststroke dysphagia: a pilot double-blinded randomized-controlled trial. Int. J. Rehabil. Res. 42, 133–138. 10.1097/mrr.0000000000000336 30676426

[B13] Ünlüer N. Temuçin Ç. M Demir N. Serel Arslan S. Karaduman A.A. (2019). Effects of low-frequency repetitive transcranial magnetic stimulation on swallowing function and quality of life of post-stroke patients. Dysphagia 34, 360–371. 10.1007/s00455-018-09965-6 30603800

[B14] Wen X. Liu Z. Zhong L. Peng Y. Wang J. Liu H.. (2022). The effectiveness of repetitive transcranial magnetic stimulation for post-stroke dysphagia: a systematic review and meta-analysis. Front. Hum. Neurosci. 16, 841781. 10.3389/fnhum.2022.841781 35370584 PMC8967953

[B15] Winstein C. J. Stein J. Arena R. Bates B. Cherney L. R. Cramer S. C.. (2016). Guidelines for adult stroke rehabilitation and recovery: a guideline for healthcare professionals from the American Heart Association/American Stroke Association. Stroke 47, e98–e169. 10.1161/str.0000000000000098 27145936

[B16] Xie Y. L. Wang S. Jia J. M. Xie Y. H. Chen X. Qing W.. (2022a). Transcranial magnetic stimulation for improving dysphagia after stroke: a meta-analysis of randomized controlled trials. Front. Neurosci. 16, 854219. 10.3389/fnins.2022.854219 35527818 PMC9072781

[B17] Xie Y. L. Wang S. Xie Y. H. Chen X. Wang Y. X. Wu Q. (2022b). Commentary: The effect of repetitive transcranial magnetic stimulation on dysphagia after stroke: a systematic review and meta-analysis. Front. Neurosci. 16, 832280. 10.3389/fnins.2022.832280 35527813 PMC9074385

[B18] Zhu Y. Gu L. (2022). Noninvasive brain stimulation for poststroke dysphagia: a meta-analysis for randomized controlled trials. Eur Neurol 85, 31–38. 10.1159/000518211 34702793

